# Micro-Scale Shear Kneading—Gluten Network Development under Multiple Stress–Relaxation Steps and Evaluation via Multiwave Rheology

**DOI:** 10.3390/polym14040846

**Published:** 2022-02-21

**Authors:** Leonhard Maria Vidal, Andre Braun, Mario Jekle, Thomas Becker

**Affiliations:** 1 Research Group Cereal Technology and Process Engineering, Institute of Brewing and Beverage Technology, Technical University of Munich, 85354 Freising, Germany; tb@tum.de; 2 Anton Paar Germany GmbH, 73760 Ostfildern, Germany; andre.braun@anton-paar.com; 3 Department of Plant-Based Foods, Institute of Food Science and Biotechnology, University of Hohenheim, 70599 Stuttgart, Germany; mario.jekle@uni-hohenheim.de

**Keywords:** dough development, shear kneading, rheology, wheat dough, protein network

## Abstract

To evaluate the kneading process of wheat flour dough, the state of the art is a subsequent and static measuring step on kneaded dough samples. In this study, an in-line measurement setup was set up in a rheometer based on previously validated shear kneading processes. With this approach, the challenge of sample transfer between the kneader and a measurement device was overcome. With the developed approach, an analysis of the dynamic development of the dough is possible. Through consecutive stress–relaxation steps with increasing deformation, a kneading setup in a conventional rheometer is implemented. Fitting of the shear stress curve with a linearization approach, as well as fitting of the relaxation modulus after each kneading step, is a new way to evaluate the matrix development. Subsequently, multiwave rheology is used to validate the kneading process in-line. The shear kneading setup was capable of producing an optimally developed dough matrix close to the reference kneading time of 150 ± 7.9 s (n = 3). The linearization approach as well as the power-law fit of the relaxation modulus revealed gluten network development comparable to the reference dough. With this approach, a deeper insight into gluten network development and crosslinking processes during wheat flour dough kneading is given.

## 1. Introduction

The most widespread usage of wheat flour is as kneaded dough, as it is consumed as processed food in the form of baked goods. Through mechanical energy input and hydration, wheat flour–water mixtures produce viscoelastic dough matter. The wheat dough owes its unique viscoelastic properties to its crosslinked protein network [1] and enables a variety of food applications. In the beginning of the dough kneading procedure, the mechanical energy input together with the hydration of the flour particles causes a crosslinking of glutenins and gliadins to a continuous protein network. This network is known as the continuous gluten phase [2,3,4]. The basis for all baked goods is the production of an optimally developed gluten phase in the dough matrix. Therefore, the optimum consistency of a dough is achieved when the dough best withstands the deformation of the kneading geometry. This development stage of the dough matrix is often indicated by the mentioned energy maximum in a torque-recording kneading machine and is therefore the peak of dough consistency [5]. The gluten phase also embeds starch granules, which promote internal friction during kneading. In addition, small gas nuclei are introduced during kneading and are evenly distributed in the dough matrix. These nuclei then serve as starters for the crumb evolution during proofing and baking.

The main task of kneaders in dough mixing is the transfer of a given amount of mechanical energy into the material to transform and develop it to the optimum consistency [6]. For the incorporation of the crucial energy input into the gluten network in wheat dough, different types of kneaders are available. For all kneaders, the transferred mechanical energy comprises tension, compression and shear to the forming dough [7]. Depending on the kneader geometry, rigid body rotation also takes place during dough movement in the kneaders. It is difficult to accurately define the crucial energy for the optimum development point of dough in general for all kneader types. Moreover, it is not clear what type of deformation is responsible for the ongoing crosslinking processes in the matrix. In industrial dough production, the rotating blades of high-speed mixers transfer energy predominantly by shear [7], whereas spiral hook kneaders incorporate crucial energy into a dough mostly by tension and compression [8,9]. According to Peighambardoust et al. [10], a total energy input of approximately 30 kJ/kg is required to produce an optimum wheat dough, but depending on the flour type and the mixer used, the required energy input can reach up to 100 kJ/kg. As suggested by Anderssen et al. [11], the evolving rheological behavior of the kneaded material will not be determined only by its current configuration, but also by the type, magnitude and duration of the applied forces to which it is subjected. With respect to these circumstances, the kneading process is difficult to reproduce, because it is so highly complex that as its composed of several force effects (as described above). As a result, it is hardly possible to completely trace the development of the dough matrix analytically. With regard to these barriers, a measuring device is missing that develops a dough matrix and additionally offers the possibility of in-line analysis. A farinograph or DoughLAB provides this approach, but with the major limitation of complex kneader geometry. In addition to the complexity limitations, interruption and sample transfer of the kneaded matter into additional equipment (e.g., rheometer, Kieffer rig) is mandatory to evaluate the dough matrix at different stages of development. Therefore, the possibility to measure the network formation in-line through a shear rheological approach in relation to the process parameters (energy input, temperature) and ingredients composition (flour type, water addition, other ingredients) would overcome this analytical gap. With this objective, several shear techniques have already been used to investigate and explain the rheological evolution of dough under shear load and their effects and changes during the development and processing of dough. As a result of this approach, [12] first succeeded in shear mixing dough in a special apparatus other than an extruder. Tietze et al. [13] introduced a shear mixing method directly in a conventional rheometer with shear in one direction. In the literature, it is found that shearing in one direction can produce dough-like structures but at the same time causes a separation of gluten and starch in the geometric cross-section [10,14,15,16]. The previous works applied simple one-directional shear at various speeds. A determinationof optimum development of the dough matrix in these works was difficult, as the process was affected by the separation processes. With the application of multiple stress–relaxation steps, this method was improved in Tietze et [7], as the phase separation was suppressed. The determination of the dough development in the previous work of Tietze et al. [7] was evaluated from mechanical relaxation spectra calculated from multiple stress–relaxation steps. The distribution of relaxation times then provided information about dough development. This dough development evaluation was an important step towards in-line rheological analysis systems. However, these spectra required a large computational effort, and the system could not be fully rheologically validated in-line. In other works, a multiwave frequency sweep method was evaluated on dough, which resulted in immense time savings in measurements [17,18]. This method allows the frequency sweep measurement to be performed during short interruptions in kneading or during the relaxation phases. With the possibility of additional in-line rheological frequency sweep testing in an improved shear kneading setup, the informative value could be expanded significantly.

The aim of this study was to develop an optimized shear kneading technique that produces an optimally developed wheat dough matrix in a rheometer and to evaluate its mechanical behavior compared to conventional kneaded dough in-line. Without a sample transfer to other devices, the system provides all necessary data to determine the stage of dough development without high computational effort. The developed method contains consecutive stress–relaxations steps to incorporate the crucial mechanical energy to form a dough, as well as time-efficient multiwave frequency sweeps. With the multiwave frequency sweep testing as an additional evaluation of the stress–relaxation results, a distinct insight into the network evolution processes and changes in the dough matrix should be enabled. The implemented in-line frequency testing gives a direct comparison to the different stages of network development of conventional kneaded dough. Without sample transfer or interruption of the kneading process, a gain of knowledge regarding energy-dependent dough matrix development is achieved. With this aim, the new method is capable of producing wheat flour doughs in a rheometer for further rheological investigations at very small scales. Resulting from this, the shear kneading technique is capable of analyzing the smallest batches and sample sizes as are usual in breeding or flour blending applications.

## 2. Materials and Methods

### 2.1. Development of the Wheat Dough Matrix

German commercial wheat flour type 550 with 14.34 ± 0.24 g moisture per 100 g flour (AACCi 44-01), a protein content of 11.78 ± 0.05 g per 100 g dry flour (AACCi 46-16, N × 5.7), 0.53 ± 0.01 g ash per 100 g dry flour (ICC 104/1) and 26.18 ± 0.12 g wet gluten per 100 g flour was used in this study. Dough resistance and water absorption were measured in a Z-kneader DoughLAB (Perten Instruments AB, Hägersten, Sewden) according to AACCi 54–70.01 to determine the required dough development time (DDT).

### 2.2. Fundamental Shear Rheology 

All rheological measurements were carried out in a MCR502 rheometer (Anton Paar, Ostfildern, Germany) with parallel plate geometries. To maintain constant humidity and temperature, a CTD 180 Humidity Ready (Anton Paar, Ostfildern, Germany) chamber was set to 30 °C and 80% relative humidity for all trials.

#### 2.2.1. Dough Produced in a Z-Kneader

For the shear rheological measurements of classical kneaded dough, 50.2 g flour (corrected to 14% moisture) and 28.10 mL demineralized water were kneaded at 63 rpm using a Z-kneader equipped with a 50 g mixing bowl. After kneading, 4 g of dough was placed centered between cross-hatched parallel rheometer plates (25 mm diameter). Once reaching the constant gap of 2 mm, all excess dough was removed, and the cut surface was coated with paraffin oil to prevent dehydration. 

#### 2.2.2. Shear-Kneaded Dough

**Figure 1:**
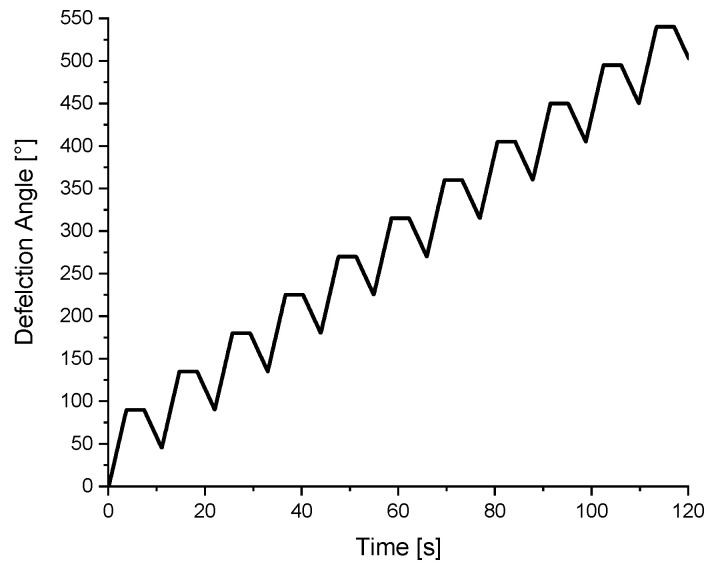
Increase in deflection for the kneading procedure with implemented relaxation phases.

To produce shear-kneaded dough in the rheometer, a plane plate–cylinder geometry setup with 25 mm diameter of the upper plate geometry and 25.1 mm inner diameter of the cylinder was used. For all experiments, 192 mg of flour and 108 µL of demineralized water, to match the water dosage of the Z-kneader, were kneaded in the rheometer according to the following procedure. The gap was set at a height of 650 µm to obtain a completely sample-filled geometry. The deformation was applied in an oscillatory matter with an asymmetric deflection angle back and forth. After each 90° forward deformation, a 4 s relaxation step was implemented (see Figure 1), followed by a 45° reverse deformation. 

### 2.3. Evaluation of the Dough Matrix Development 

#### 2.3.1. Frequency Sweep Testing 

After the resting of the Z-kneaded dough in the measuring gap, a frequency sweep was performed in a range from 0.1 to 50 Hz at a constant deformation of 0.05%. The obtained complex shear modulus (G*) data were fitted according to Gabriele et al. [19] with a power law equation (c.f. Equation (1)).

(1) $$\left|G^{*}\right|=A_{f}\times \omega^{\frac{1}{z}}$$

where *ω* is the angular frequency (s^−1^), *A_f_* refers to the network strength (Pa s^1/*z*^) and z refers to the network connectivity (–) [19,20,21,22].

#### 2.3.2. Shear Kneading Setup 

One approach to evaluating the ongoing network evolution was a fitting of the exponential decrease in the linear relaxation modulus *G*(*t*). The relaxation modulus is calculated from the applied shear stress at time t, *σ*(*t*) and the shear strain at the beginning of the relaxation *γ*_0_. The fit calculates for each relaxation step as follows [23] (c.f. Equation (2)):

(2) $$G\left(t\right)=\frac{\sigma \left(t\right)}{\gamma_{0}}=S\times t^{-r}$$

where *S* refers to the stiffness of the matter and *r* is the relaxation exponent. 

As *G*(*t*) is dependent on deformation/shear strain *γ*, another approach to evaluating the network evolution and kneading performance of the shear kneading setup focusing only on geometric properties and measured torque was considered. In fitting the shear stress decrease during the relaxation step, the behavior of the matter was evaluated according to Bhattacharya or Peleg [24,25]. This was achieved with a linearization of the exponential decreasing shear stress (c.f. Equation (3)).

(3) $$\frac{\sigma_{0}\times t}{\sigma_{0}-\sigma \left(t\right)}=k_{1}+k_{2}\times t$$

In this normalization, *σ*_0_ is the shear stress at the beginning of the relaxation step and *σ*(*t*) the respective shear stress at time *t*. Calculated from the linearization parameters *k*_1_ and *k*_2_, it follows that 1/*k*_1_ is the initial decay rate and 1/*k*_2_ denotes the asymptotic value of the relaxed portion of the initial stress. It is stated that 1/*k*_2_ goes towards 0 for an elastic solid and 1/*k*_2_ goes towards ∞ for a liquid [25].

#### 2.3.3. Multiwave Frequency Sweep Testing of Shear-Kneaded Dough 

In an additional shear kneading setup, multiwave frequency sweeps were performed after the relaxation steps at specific setpoints of the shear kneading process to characterize the state of the dough. The framework of multiwave instead of standard frequency sweeps was chosen due to the shorter measuring time. The results from the multiwave tests on dough were carefully compared with the standard frequency tests to guarantee a correct rheological characterization of the dough’s state. 

The fundamental frequency was ω_0_ = 1 Hz with an amplitude of 0.05%. The harmonic frequencies are each a multiple of the fundamental frequency, namely ω_1_ = 3 Hz, ω_2_ = 4 Hz, ω_3_ = 5 Hz, ω_4_ = 6 Hz, ω_5_ = 7 Hz, ω_6_ = 8 Hz, ω_7_ = 9 Hz and ω_8_ = 10 Hz, each with an amplitude of 0.05%. The resulting peak amplitude was 0.115%. The amplitude and harmonics were chosen to be in the range of linear viscoelasticity of dough networks.

The obtained sweep data were fitted accordingly to 2.3.1 with the power law equation from [19].

#### 2.3.4. Energy Consumption 

To evaluate the specific mechanical energy (SME) needed to develop the dough matrix at specific kneading times, it was calculated from the measured torque according to [14] (cf. Equation (4)):

(4) $$SME=\frac{\omega }{m}\;\times \int M_{d}dt$$

In this equation, *ω* (s − 1) is the rotational speed, *m* (kg) is the mass of the kneaded material and *M_d_* is the torque (N m) measured on the kneading arm. The *SME* (kJ/kg) was calculated between *t* = 0 and the respective kneading time td. 

#### 2.3.5. Statistical Analysis

All measurements were performed in triplicates. The standard deviation accounts for the deviation between these triplicates. Mathematical and statistical evaluation was performed using MATLAB (R2018a, MathWorks Inc., Natick, MA, USA) and Origin (2018b, OriginLab Corporation, Northampton, MA, USA). 

## 3. Results and Discussion

### 3.1. Standard Mixing Procedure in a Torque-Recording Z-Kneader

**Figure 2:**
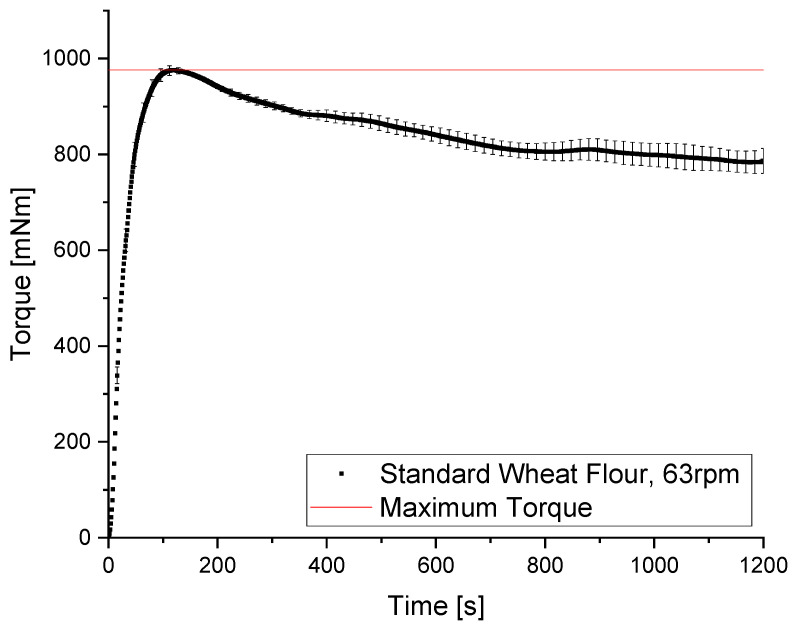
Kneading curve for standard wheat flour measured in a torque recording Z-Kneader (DoughLAB). Maximum peak resistance is marked at 976 mNm. Means are shown with standard deviation (n = 3).

A torque-recording Z-kneader was used to determine the time to maximum resistance of the evolving dough matrix against the applied deformations during the kneading process. The kneading process can be divided into hydration and distribution, crosslinking and network development and, after a certain point of applied load, network breakdown. In the beginning of the kneading process, the protein gets hydrated, and the contained glutenins unfold and then interconnect [26]. Within this polymeric glutenin network, a fibrillary gliadin network emerges with ongoing kneading, which behaves as anisotropic elastomers with an elastic modulus that can be the same as that of fibrous elastin (depending on the degree of hydration) [27]. This gives the dough matrix its unique viscoelastic attributes. The backbone of the gluten network consists of covalent disulfide bonds, which contribute to the plasticity of dough. On the other hand, it contains elasticity-determining non-covalent interactions, especially intra- and intermolecular hydrogen bonds, which lead to the formation of loops and trains in the network structure, as proposed by [28,29]. The typical torque vs. time curve in Figure 2 shows the prominent peak value of the recorded torque after 150 ± 7.9 s (n = 3) kneading time (dough development time, DDT). After reaching the DDT, a short stability phase can be observed, followed by a decrease in the measured torque due to an ongoing network breakdown. After reaching the maximum, a wheat dough is overmixed, and a higher degree of rupture of the protein matrix can be observed [30]. This overmixed phase was observed with ongoing kneading over 225 s.

Besides the DDT and the following short stability phase up to 190 s, additional setpoints for rheological investigations were chosen to evaluate the network development throughout the kneading process. The first point sets the lower kneading time limit at which homogeneous matter is first reached, and with this achieved, the rheological investigation is reliable. In addition to 50, 100 and 150% DDT (see Table 1), the end of the kneading stability at 190 s was chosen. To get insights into overmixed dough behavior as well, two stages at 660 s and 1200 s were also chosen. 

**Figure 3:**
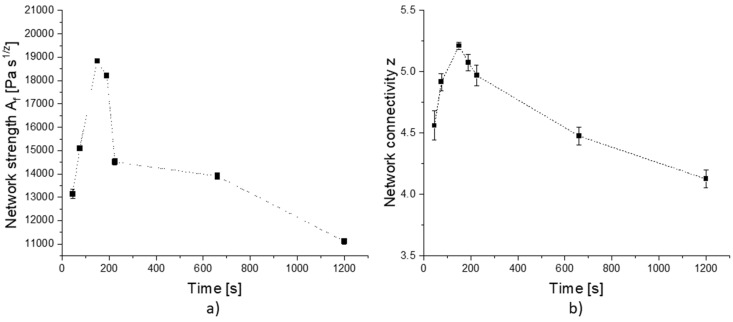
(**a**) Fitting parameter Af and (**b**) parameter z from power-law fitting the frequency sweep results of dough produced in a Z-Kneader according to Gabriele et al. [19]. Means are shown with standard deviation (n = 3).

To quantify the strength of short-range interactions as they occur in starch–gluten or starch–starch polymers, a frequency sweep in a rheometer with a low deformation within the linear viscoelastic region was used [26]. Along the dough matrix evolution, the strength of the network-specific short-range interactions was quantified considering the network strength *A_f_* and network connectivity *z*. To obtain these coefficients, the power law equation was used to fit the frequency dependency of the complex shear modulus G* [19,31,32,33,34]. For this purpose, a dough sample was taken after each step and analyzed using a frequency sweep. As shown in Figure 3, the rheological behavior of wheat flour–water dough follows the trend of the kneading curve mentioned before. At 100% DDT, both parameters reach a peak value, which drops when the dough gets over-kneaded and the network starts to break down. After reaching the end of the stability region (190 s), a drop in the network strength is clearly visible and even more pronounced than the drop in the connectivity. This drop could be explained by the breaking of disulfide bonds and a release of free water into the matrix [35]. Due to the broken covalent bonds, the non-covalent bonds are more pronounced, which explains the slight connectivity decrease, as they are weaker. The more connected but weaker bonds of smaller polymer compounds cause a slow decrease in the parameter *z* (-). As mentioned in Don et al. [36], the quantity of large protein clusters, but not the protein concentration itself, changes, and so the network configuration changes to a fibrillary appearance with less strong but highly connected protein strands. 

### 3.2. Shear Kneading with Increasing Deformation

**Figure 4:**
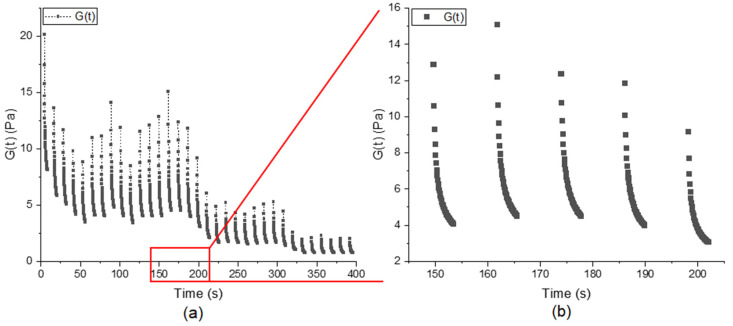
(**a**) Decrease in *G*(*t*) during the relaxation steps for standard wheat flour and water [n = 1] as an example of the shear kneading process over the whole 400 s kneading process. (**b**) Zoom in on the exponential decrease in values for the relaxation steps from 150 s to 200 s kneading time.

To produce dough in the rheometer, the shear kneading setup (see Section 2.2.2) was used. With the ongoing deformation/kneading in the rheometer gap, a dough matrix was able to be developed. In Figure 4, the decrease in the relaxation modulus after each positive 90° deflection is shown. The level of relaxation varies with ongoing kneading time. For each relaxation step, another magnitude of the decreasing *G*(*t*) value is measured. In addition to that, differences in the relaxation speed of the dough matrix can be observed. This relaxation step is important for network formation, as it enables interconnection during recoil and gluten strand relaxation. With this relaxation, the material is able to reduce the inner shear-induced tension, and protein recoil takes place [37]. These kneading pauses, together with the gradually increasing deformation due to the asymmetric deflection of the rheometer geometry, enhance the interconnection and relaxation processes in the dough matrix and support crosslinking throughout the whole sample cross-section. This varying relaxation behavior was utilized to evaluate the dough matrix development according to Equation (2). The ‘gel strength’ parameter (*S*) from the power law gel model is an appropriate descriptor for both the linear and non-linear viscoelasticity of wheat flour doughs generated using a wide range of formulations. The calculated relaxation exponent n gives no deeper insight into dough matrix properties, as it shows no correlations between linear and non-linear dough matrix properties [22]. 

**Figure 5:**
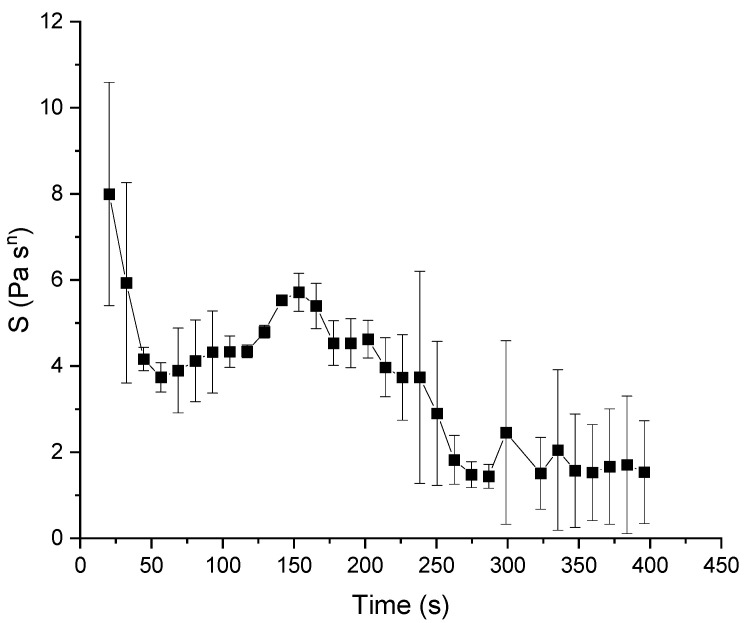
Gel strength *S* (fit of the relaxation modulus according to Bhattcharya et al. [24]) calculated from the relaxation steps during the shear kneading process of standard wheat flour and water. Means are shown with standard deviation (n = 3).

The ‘gel strength’ ***S*** depends on the mobility of the chain segments and is determined by the persistency length and crosslink density [38]. The relaxation exponent ***r*** may have values in the range 0 < r < 1. The calculated results ranged between 0.262 ± 0.003 and 0.176 ± 0.002, which was in good agreement with the literature. Sun et al. [22] stated that for the recovery phase, very good fits with the power law were obtained, except at long relaxation times. Therefore, the approach of fitting the short-time relaxation in the hold phase is very promising, as it is sufficient to describe the dough matrix properties even when the relaxation exponent is not at a plateau value at the end of the relaxation step. As shown in Figure 5, at first, a decrease in the values of ***S*** around 4 Pa × S^r^ can be observed. Before reaching this state, mainly flour particle–particle interactions are dominant in the matter and stand in a competitive way with the evolving network, which is the reason for the higher first two start values [39]. These high values with high standard deviations at the beginning of the kneading can be explained by visual observed inhomogeneities of the sample (data not shown) and the before-mentioned particle–particle interactions. After reaching the DDT from the DoughLAB around 150 s and after reaching farinograph stability (around 190 s), a decrease to a low level, around 2 Pa × S^r^, was observed in the gel strength. This observation covers the assumption that at the DDT, the dough behaves like a weak physical gel [19,22]. With the implemented shear kneading technique, a dough matrix development in a time region similar to the DoughLAB DDT was reached. This curve shows an optimum state at the desired DDT when the network interactions become stronger than the particle–particle interactions, indicating the presence of a well-developed network structure. Stress–relaxation measurements at small strain amplitudes (0.1%) carried out for different doughs with different strengths from Safari-Adri et al. [40] showed that doughs with different strengths showed no difference in their relaxation behavior. However, at a range of large strains > 29%, the relaxation behavior was better correlated with the strength of dough [41]. Thus, the large deformations applied in the used rheo-kneading setup should be sufficient to determine the dough strength with respect to evaluating the optimum development stage of the matrix. Therefore, the results obtained from the stress–relaxation kneading, with a set strain in the process of 650%, are in good agreement with the necessary large stains.

**Figure 6:**
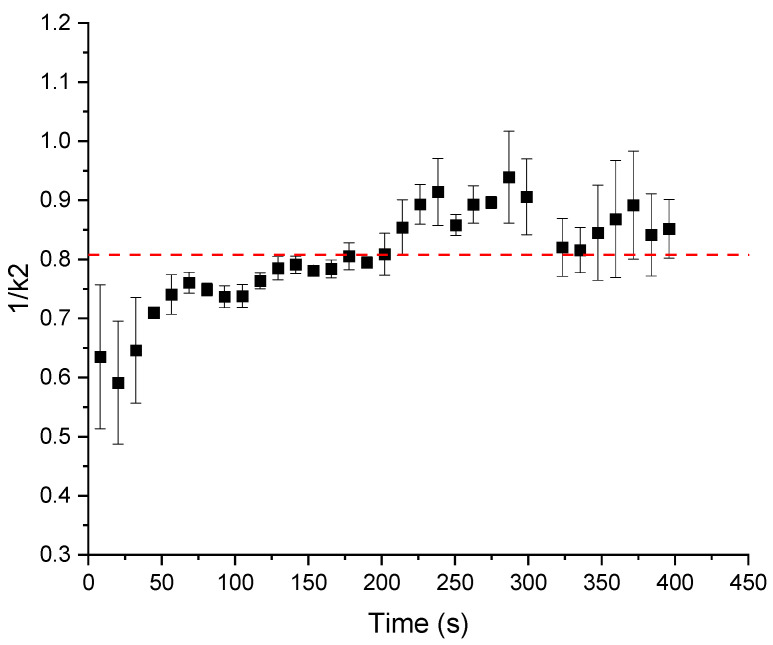
Parameter 1/k_2_ calculated from normalizing the shear stress curves of the shear kneading process of standard wheat flour and water. Means are shown with standard deviation (n = 3).

Following another data evaluation approach with the normalization of the measured shear stress curves for each relaxation step during the rheo-kneading with Equation (3) [24,25], Figure 6 shows the calculated parameter 1/*k*_2_. The graph shows the increase in the reciprocal value *k*_2_ over the kneading time. The horizontal red dashed line is the standard value for 100% DDT dough from a Z-kneader. To obtain this reference value of 0.81, the optimally developed dough was transferred to the rheometer and shear-kneaded for three consecutive shear kneading steps in a PP-smooth geometry. Since dough behaves as both a viscoelastic liquid and solid, this standard value is in good agreement with the behavior of optimally developed dough [42]. As shown in the increase in the values of 1/k_2_ in the first sample homogenization and hydration, the network’s evolution and stabilization take place until approximately 200 s. During hydration, glutenin proteins unfold and interconnect to a continuous network. Within these interconnected protein strands, the globular gliadins form a fibrillary network [26] which can be observed with the increasing viscoelastic behavior of the matter. Starch gets embedded in the system and, together with the gliadins, viscosify the sample’s flow behavior after homogenization and hydration. After reaching the over-kneaded state, the matter becomes more viscous and loses its viscoelastic properties. This over-kneaded state is reached at the vertical red dashed line. The increasing values after reaching the over-kneaded stage indicate a network breakdown and the viscosization of the dough matrix. With the values close to 1, the behavior of the dough approaches a more liquid state. After reaching the over-kneaded state, a non-constant material response causes the high standard deviation (SD) values after 200 s (as shown in Figure 6, the SD goes up for over-kneaded dough). The calculation of the consumed mechanical energy in the shear kneading reaches 10.27 ± 0.81 kJ/kg until 150 s kneading time (at 400 s, *SME* equals 27.95 ± 6.79 kJ/kg). For classical Z-kneaded and extruded doughs, a range from 30 to 100 kJ/kg to produce a fully developed dough matrix is customary [13,43,44,45]. The low value of the *SME* can be explained by the incorporation of only shear deformations and thus a lower amount of dissipating energy through other deformations than shear within the kneading process.

### 3.3. Multiwave Evaluation of the Shear Kneading Setup with Increasing Deformation 

For further evaluation of the kneading process and dough matrix development, multiwave frequency testing was implemented in the shear kneading process. 

**Figure 7:**
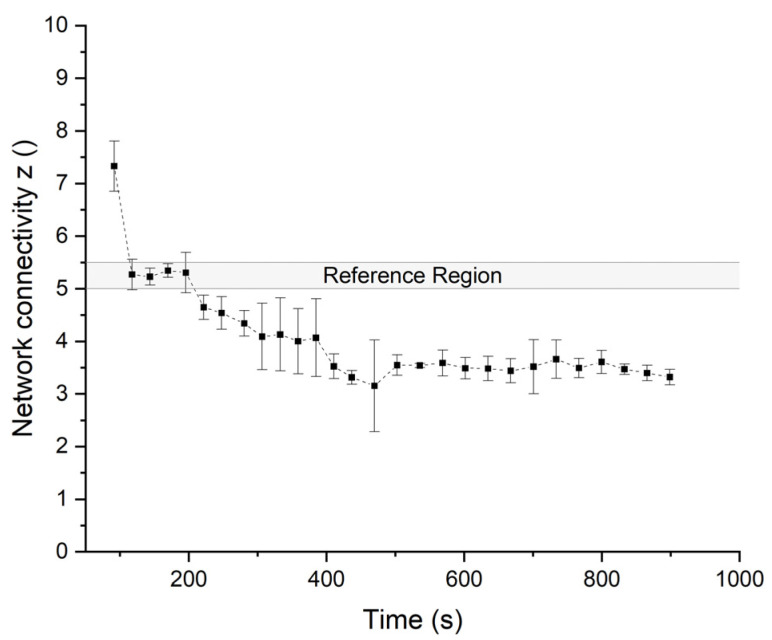
Network connectivity z from power-law fitting G*. Measured in an additional test setup via multiwave frequency sweeps after certain relaxation steps during stepwise increases in shear kneading. The reference region (z = 5 to 5.5) shows the range of values of a Z-kneaded dough at its dough development time (150 s). Means are shown with standard deviation (n = 3).

In Figure 7, the network connectivity *z* is obtained from power-law fitting the G* values of the multiwave measurements with the reference region obtained from measuring Z-kneaded dough at the predetermined DDT. Before the hydration and homogenization of the matter, the measured partly hydrated flour particles show a higher degree of the calculated network connectivity. These values can be classified as initial noise due to visual observed inhomogeneity of the sample, as mentioned before (data not shown). This behavior is explained by findings from Fröhlich et al. [39] for polymer networks with filler particles comparable to dough (containing the continuous gluten phase and starch particles as types of fillers). For the partially unhydrated flour particles in the early kneading stage and the contained starch granules, the filler–filler and filler–polymer interaction is competitive in a certain way. After adding the filler, at low strains, the modulus rises more than the high-strain modulus, resulting in a non-linear viscoelastic behavior, known as the Payne effect. The stiffer behavior at the unhydrated stage of the dough at the beginning of the kneading is explained by the fact that the filler cannot be deformed. In this early stage, partially unhydrated flour particles and starch are in the rigid filler phase. From the second multiwave sweep, the stress softening after homogenization and hydration is attributed to the breakdown of the inter-aggregate association respective to the breakdown of the filler network. In this case, the breakdown of the flour particle–particle and starch–starch interactions, as well as the evolution of a continuous polymeric network after homogenization, takes place. This equals approx. 120 s kneading time with a 900° deflection and therefore 2.5 revolutions of the upper geometry. According to the critical gel theory of Winter and Chambon [46] as basis for the fitting of $\left|G^{*}\right|$ and the assumption of Gabriele et al. [19] that no changes in the network nodes or strands take place during the frequency sweep, the first measured value can be seen as the earlier-mentioned noise, but it also shows the successful evolution of the dough in the ongoing kneading process. After over-kneading, at approx. 200 s, the values for the network connectivity *z* (-) drop below 5 and show a destruction of the protein network. Due to the small sample size and the high shear forces within the geometry gap of the rheometer during kneading, the network shows inconsistent attributes between 300 s and 400 s kneading. After a nearly complete breakdown of the network after 500 s kneading time, the SD gets smaller, as the network properties then equalize.

## 4. Conclusions

To solve a major challenge in the evaluation of dough development, namely sample transfer from the kneaders to the measuring instruments, a shear kneading technique was investigated in a conventional rheometer. Based on fundamental rheology testing, the evolution of the continuous gluten phase in the developing dough matrix could be verified with in-line multiwave rheology tests. Additional to previous works on shear kneading techniques [7,10,13,14], the improved kneading process with the non-interrupting in-line rheology testing enabled the dough matrix evolution to be mapped from the very beginning of the kneading process. Therefore, the comparability of shear-kneaded to classically (in a conventional Z-kneader) produced wheat flour dough along the kneading process could be shown. The shear kneading setup in a conventional rheometer represents a useful tool to analyze structural formation reactions of the gluten phase, as well as a controlled energy input method to investigate the influence of deformation on network evolution processes. 

In this study, the applicability of the applied shear stress–relaxation steps to develop a dough matrix was shown. The initial particle–particle interactions of the non-hydrated flour and starch granules were rheologically comprehensible in the early stage of dough development. For the evolving dough matrix, changes in flow and relaxation behavior, as well as gel strength increase due to ongoing hydration and homogenization, were observed. For the evolution of a continuous gluten phase, the applied shear forces were sufficient to develop the dough matrix over the whole sample cross section. Typical for kneaded wheat flour doughs, an optimum development stage was observed that was close to the externally (in a DoughLAB) determined one. Therefore, the identification of the crucial energy to develop the gluten phase during kneading became more comprehensible since having only one deformation type eased the calculation. As the results show, the energy consumption was comparable to other types of kneaders, except that no energy was wasted in the parallel plate rheometer due to the small sample size and large contact area of the kneading geometry. This step toward a fully comprehensible energy transfer to the kneaded sample is elementary for the understanding of microstructure formation. Since the results are carried out only for native wheat flour, the adaption of the kneading technique to other gluten-containing samples must be further investigated. Additionally, the limitation of the validity of the multiwave frequency sweep to the previous determined linear viscoelastic region for the kneaded sample sets a limit to the frequencies and amplitudes that can be measured simultaneously. Nevertheless, the developed kneading technique provides the basis for small-scale investigations of dough behavior during further processing, e.g., gas expansion during the proofing and baking steps.

## Figures and Tables

**Table 1:** Selected kneading times to evaluate the network evolution along the ongoing kneading process of standard wheat flour water dough.

Reaching 400 FU	50% DDT	100% DDT	End of Stability	150% DDT	Over-Kneaded 1	Over-Kneaded 2
45 s	75 s	150 s	190 s	225 s	660 s	1200 s

## Data Availability

The data presented in this study are available on request from the corresponding author.
